# Screening of Humic Substances Extracted from Leonardite for Free Radical Scavenging Activity Using DPPH Method

**DOI:** 10.3390/molecules27196334

**Published:** 2022-09-26

**Authors:** Attila Csicsor, Etelka Tombácz

**Affiliations:** 1 Doctoral School of Environmental Sciences, University of Szeged, Rerrich Béla tér 1., H-6720 Szeged, Hungary; 2 Hymato Products Ltd., Kossuth u 33., H-8225 Szentkirályszabadja, Hungary; 3 Soós Ernő Water Technology Research and Development Center, University of Pannonia, H-8200 Veszprém, Hungary

**Keywords:** humic substances, antioxidant, humic acid, fulvic acid, himatomelanic acid, leonardite, radical scavenging activity, DPPH, extraction

## Abstract

Humic substances (HSs) have been researched for a long time and still manage to surprise humanity today. According to the latest research, in addition to their previously well-known effects, they also have antioxidant properties. However, this previous research does not examine the difference in the antioxidant effect of the fractions extracted/produced in different processes; they do not consider the light absorption of the HSs, which falsifies analysis based on the measurement of color change over time. In the present work, HS fractions were obtained from leonardite, the extraction processes can also be implemented on an industrial scale. The fractions were characterized by elementary analysis, UV–Vis and FT-IR spectroscopies, to prove that our self-extracted samples have similar characteristics to the International Humic Substances Society (IHSS) standard samples. The different methods of HS fractionation affected the elemental composition, and the spectral characteristics. The antioxidant effect was investigated using the DPPH method to screen the antioxidant efficiency of humic, fulvic, and himatomelanic acids. In addition, we compared our results with the IHSS standard samples to obtain a more comprehensive picture of the antioxidant effect of HSs extracted in different ways according to the DPPH method. Based on our results, the extraction method affects not only the physico-chemical properties but also the free radical scavenging activity of the fractions.

## 1. Introduction

As reported by the International Humic Substances Society (IHSS), humic substances (HSs) are complex and heterogeneous mixtures of polydisperse materials. They form in soils, sediments, and natural waters by biochemical and chemical reactions during the decay and inherent transformation of plant and microbial remains in a process called humification [1]. HSs are major components of the natural organic matter (NOM) in soil and water as well as in geological organic deposits such as lake sediments, peats, brown coals, and leonardite.

According to the operational definition of IHSS, HSs can be divided into four fractions of different solubility. Such as humin, humic acids (HA), fulvic acids (FA), and alcohol-soluble himatomelanic acids (HY). Each fraction has different characteristics; they do not have a uniform molecular structure, and only the functional groups of molecules behave similarly [2]. It is important to note that the exact composition of HSs differs depending on their origin (soil, peat, leonardite, water, or air), location, and type of extraction technology. The fractions may have different properties due to differences in composition, and they may contain distinct functional groups or similar functional groups in various proportions. There are several types of methods for the separation of HS fractions. The methods of IHSS give one of the purest fractions; however, the procedures are complicated, multi-stepped, expensive, and difficult to implement in industrial quantities. One of the goals of our research is to produce fractions that can be extracted on a larger, industrial scale. We were also interested in the effects of the fractionation processes on two large groups (alkaline and acidic) of extraction, how they change the composition of the fractions, and, above all, the antioxidant properties of the fractions. It is important to note that the fractions were not examined for food industry safety purposes; therefore, the extraction procedures presented here do not ensure the separation of the samples from a food safety point of view.

Because of this wide diversity of HSs, they are used in almost every agricultural field. Studies indicate that using HSs can stimulate plant growth and oxygen transport, accelerate respiration and seed germination, and promote the efficient utilization of nutrients by plants [3]. Using HSs in animal husbandry has many advantages; it can improve nutrient absorption, accelerate digestive functions that can regulate growth, and influence the metabolism resulting in an antiviral or antibacterial effect [4]. Many studies have shown that HSs are able to prevent inflammation, stimulate the immune system, and absorb toxic materials [5].

As far as we know, HSs have been used for at least thousands of years for human applications. Based on much research, HSs have been found to possess antiviral, anti-inflammatory, heavy metal chelating, antimutagenic, antitumor, antioxidant, and photo-protective properties [6]. These properties are linked to the HSs’ cellular and physiological effects such as regulating genes or ion exchange, modulating stress reactions, and activating different signal transduction processes [7]. Free radical-related diseases (e.g., obesity, cardiovascular disease, and malignancies) still play a major role in human life around the world, and HSs could be a good alternative to prevent these diseases [8].

Every molecular species that is capable of independent existence and contains an unpaired electron is called a free radical. Antioxidants are the main free radical scavengers, most likely reactive oxygen species (ROS) and reactive nitrogen species (RNS) scavengers. These free radical scavenging molecules are able to delay, reduce, or inhibit the radicals’ cellular damaging capacity [9]. The question may arise as to why HSs would have an antioxidant effect. The first reason is that they are mostly formed from plant residues and plants are able to produce antioxidant molecules. These molecules over time build into the structure of HSs partly or fully. Second, the chemical structure of HSs is able to answer this question. Considering their chemical structure, they are polyhydroxy carboxylic acids with quinone and semiquinone groups. In some aspects, they are similar to flavonoid phenols, in which the so-called flavone skeleton is polysubstituted by hydroxy groups. However, they also have a quinoid structure that is known to be responsible for antioxidant properties [10]. These properties of HSs have already been demonstrated in a number of scientific publications by both classical analytical methods (redox titrations) and instrumental analytical measurements (Electron paramagnetic resonance, ESR) [11,12,13].

There is an increasing demand for the quantitative determination of antioxidant capacity (AOC), which is why many analytical procedures and measurement systems have been developed in the past decades. AOC measurement methods can be divided into two main groups. The first group is the electron transition (e.g., 2,2-diphenyl-2-picrylhydrazyl (DPPH) radical-scavenging; ferric reducing antioxidant power-(FRAP); Cupric reducing antioxidant activity (CUPRAC)) based, and the second group includes methods based on hydrogen atom transfer (e.g., oxygen radical absorbance capacity (ORAC); photo chemiluminescence (PCL); total radical-trapping antioxidant potential (TRAP)) [14].

There are quite good articles dealing with the characterization of the antioxidant properties of HSs, measured by different methods; however, to the best of our knowledge, leonardite fractions have not been systematically investigated. *Avvakumova* et al. [15] examined HSs extracted from peloids, measuring the antioxidant activity (AOA) of the HS fractions using radical-chain-initiated oxidation of 1,4-dioxane (I) under standard conditions. *Khil’Ko* et al. [16] extracted HA from brown coal, and they studied the liquid-phase oxidation of cumene and ethylbenzene initiated by azobisisobutyronitrile (AIBN, the reaction initiator) in dimethyl sulfoxide (DMSO) solution in the presence of HA in its wide concentration range. *Zykova* et al. [17] also tried to prove with electrochemical measurements and the DPPH method that HA extracted from peat has antioxidant properties. *Karadirek* et al. [18] investigated the total AOC of HA sample obtained from different turf sources using different methods (such as CUPRAC; Folin–Ciocalteu method, FC; QUENCHERCUPRAC; QUENCHER-FC). Based on their results, each method was able to prove that HSs have AOC, but it is hard to correlate these results. *Klein* et al. [19] used the ORAC method to quantify the AOC for each of the HA and FA samples obtained from different sources (soil, peat, natural water, and coal). Their data for terrestrial sources (peat, soil) indicate that FAs were characterized by much higher AOC values compared to HA isolated from the same source. The antioxidant effect of HA can also be determined by electrochemical experiments. *Aeschbacher* et al. [20] used mediated electrochemical oxidation (MEO) to provide electron transfer from the electron-donating moieties in HS to working electrodes to obtain information on the AOC of HSs. *Verrilo* [21] used the TPC (Total Phenolic Content) method to gain some information about its antioxidant content. They found a strong correlation between the TPC of HS samples and their antioxidant capacities. Namely, that the antioxidant activities of HS samples were related to their lignin fragment content. *Smirnova* et al. [22] examined the antioxidant activity with the presence of active acidic groups (–COOH, phenolic –OH). Their results showed that HA exhibits antioxidant activity in the radical oxidation of hydrocarbons and pro-oxidant properties in the oxidation of vitamin C. *Nikolaev* et al. [23] measured standard and commercially available samples of HA (Suwannee River) and FA (Suwannee River). The AOC of samples was measured as a decrease in the absorbance at 734 nm of radical-cation ABTS ((2,2-azyno-bis-(3-ethylbenzthiazoline-6- sulfonic acid) diammonium salt) in the presence of the HA and FA samples in the range of concentrations 4–10 mg/mL. The results obtained at different pH levels showed a significant increase in HA and FA AOC with rising pH from 3.75 to 6.80.

One of the most common methods for measuring free radical scavenging activity is the DPPH method, which is based on the reduction in DPPH radicals. Therefore, we chose this method to obtain an insight into the free radical scavenging ability of our fractions and some reference samples and its correlation with the physicochemical properties of the HSs measured here. In our work, we try to compare the alkaline and acidic extraction methods of HS fractions and the antioxidant effect of the fractions for a broader and deeper understanding of HSs. The raw material we used is the so-called Leonardite, which naturally contains a high amount of HS (>70%).

## 2. Results and Discussion

### 2.1. Extractions

After the extractions, we obtained six different samples plus the three bought IHSS samples. All the sample names are included in Table 1.

### 2.2. Elemental Analysis

The elemental composition data are summarized in Table 2. The measured amounts of C, H, N, S, and O in our HA and FA samples are similar to that of the IHSS standard samples. Comparing our results with the elemental composition of hundreds of HA and FA samples, evaluated by *Rice and MacCarthy* [24], we can see that the O content of the sample is within range (for HA fractions: 7.93–56.6 wt% and for FA fractions: 16.9–55.88 wt%). We can say the same for the atomic ratio data in Table 2, since the H/C ratio of HA is between 0.08 and 1.85, that of FA is between 0.77 and 2.13; and the O/C ratios of HA is between 0.08 and 1.2, that of FA is between 0.17 and 1.19.

The general trend of the results is that the carbon content of the HA, FA, and HY fractions decreases, and their oxygen content increases. This is also clearly visible in the case of IHSS samples. However, differences can be seen when comparing self-extracted samples with IHSS samples. For the self-extracted samples, the C is somewhat lower, while the H content is a few percent higher. The percentages of N and S are roughly the same. The oxygen content of our fractions is higher than that of IHSS samples, and these values of samples from alkaline extraction are higher than that from acidic extraction.

Al-HA and al-HY contain similar amounts of C, but O is greater in the al-HY. Al-FA contains a lower amount of C, but its O content is the highest. Ac-HA has a slightly higher C content than ac-FA or ac-HY, but its O content is significantly lower. These results are in agreement with the work of Stevenson et al. [25]. Furthermore, al-HA contains less O than ac-HA, but this is not surprising, since oxygenated groups play a prominent role in metal coordination [25,26].

The results of the elemental analysis show that the acid extracted fractions have a higher nitrogen content (between 0.96 and 4.02%), than the fractions obtained by alkaline extraction (between 0.7 and 1.78%). We can also compare the values of the C/N ratios. The C/N ratio of the HA extracted from leonardite is higher (for both extraction methods) (between 34.71 and 68.35) than that of the IHSS HAP extracted from peat (C/N ratio: 17.82). This means that fractions extracted from peat have a generally higher nitrogen content. Looking at the FA fractions, most literature suggests that this fraction has a higher value for the C/N ratio than the HA, caused by the low N content, and a relatively high C content [27,28,29]. This relationship can be observed in the case of samples extracted from peat (two IHSS samples); however, it is not the case for samples extracted from leonardite; this ratio has reversed.

All FA fractions show a higher O/C ratio than the respective HA, confirming the trend observed by Stevenson et al. [25]. The O/C ratio of alkaline-extracted al-HA is higher than that of their acidic counterparts. Considering the H/C ratios of alkaline-extracted fractions, they are generally higher than that of acid-extracted samples. However, for samples from both extractions, the H/C ratio is higher than that of the IHSS standard samples.

HY fractions show generally the highest amount of O content and the lowest amount of C. Therefore, their O/C ratios are higher than that of HA and FA fractions. Considering the H/C ratios, this fraction reaches the highest value (for both extraction methods).

### 2.3. FT-IR Spectroscopy

**Figure 1:**
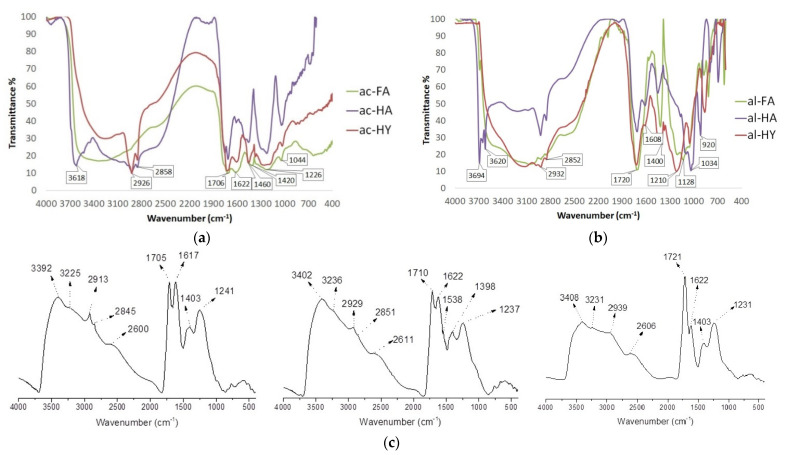
The different FT-IR spectra of the fractions are shown to compare the self-extracted samples with the IHSS samples: (**a**) acid-extracted fractions. (**b**) alkaline-extracted fractions. (**c**) IHSS standard samples from the official IHSS website: IHSS HAL (1S104H—on the left), IHSS HAP (1S103H—on the middle), and IHSS FAP (2S103F—on the right) [32].

The FT-IR spectra of samples are presented in Figure 1. They can be interpreted according to the literature data [27]. The spectra of all self-extracted samples that contain common sharp and intense bands around 1040 cm^−1^ are assigned to the C-O stretching of polysaccharides or polysaccharide-like substances and/or Si-O bonds of silicate impurities. The band at 1218 cm^−1^ assigned to C-O is the stretching of aryl ethers and phenols. A peak at 1420 cm^−1^ is an O-H deformation stretch and C-O is the stretching of phenolic OH groups. A peak at 1610 cm^−1^ can be assigned to the stretching of C=C bonds in aromatic rings, even though C=CO vibrations of conjugated carbonyl groups cannot be completely excluded at 1600 cm^−1^. The band at 1710 cm^−1^ corresponds to the C=O stretching of the carboxylic groups. The further common bands of all the three fractions are around 2850 and 2930 cm^−1^ resulting from the OH stretching vibrations of the hydrogen-bonded COOH. The FT-IR spectra show that the samples studied here differ mainly in terms of the content of inorganic constituents.

There is not too much difference in the spectra of the self-extracted samples in terms of stretching vibration. The main differences are in the fingerprint region. It can be assumed that they do not completely overlap, but they may belong to the same or very similar stretching. For example, the band at 1034 cm^−1^ appears for the alkaline samples, but it shifts to 1044 cm^−1^ for the acid samples. Each band assigned to the C-O stretching belongs to polysaccharides or polysaccharide-like substances and/or Si-O of silicate impurities. The same is true for the 1400–1420 cm^−1^, 1608–1622 cm^−1^, and 1706–1720 cm^−1^ bands; they are different for the acidic and alkaline fractions.

The differences can be found in one or two individual spectra. The band at 920 cm^−1^ can be only seen at the al-HA; it is assigned to the C-O stretching of polysaccharides. The band at 1128 cm^−1^ can be only seen in the spectrum of the al-FA fraction, which is the C-O stretching of secondary alcohols and/or ethers characteristic of the acid-extracted samples. The band at 1214 cm^−1^ was only observed in fractions al-HY and al-FA. The peaks at 2850 and 2930 cm^−1^ are not specific for the FAs but are for the other fractions. The HAs have a unique band at 3620 cm^−1^ showing the presence of kaolinite. Bands and shoulders at wavenumbers less than 700 cm^−1^ are attributed to mineral matter.

The main difference between the IHSS and our samples is that the spectra of the IHSS samples are much cleaner mainly because of the cleaner fractions due to the more precise and complicated extraction process. The fingerprint interval is not so characteristic, which also means they do not contain as many minerals as the self-extracted samples. The other difference is the bands around 3230 cm^−1^, 3400 cm^−1^ assigned to O-H stretching, N-H stretching (minor), and hydrogen bounded OH. The main similarities are the bands around 1230 cm^−1^, 1400 cm^−1^, 1620 cm^−1^, 1710 cm^−1^, the inflexion at 2600 cm^−1^ (which is very typical for HSs), 2850 cm^−1^, and 2930 cm^−1^ [30,31].

### 2.4. UV-Vis Spectroscopy

**Figure 2:**
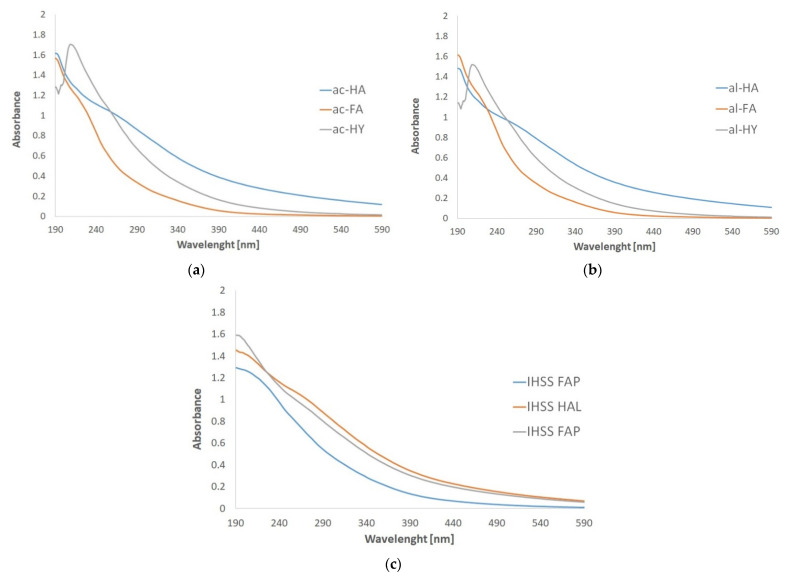
UV–Vis spectra of the different HS fractions from leonardite for comparison with IHSS samples: (**a**) the acid extracted fractions, (**b**) the fractions from alkaline extraction, (**c**) the IHSS standard samples.

UV–Vis spectra of the different fractions are seen in Figure 2. Although spectra are mostly featureless, as is usually the case with HS [29], there is a clear spectroscopic difference between the samples. The most obvious is the peak of the spectrum of the HY fractions, which is not at all typical of the other two fractions, so it seems that HY can be easily distinguished by spectroscopic tools. The spectroscopic difference between the FA and HA fractions is also obvious, while the spectrum of the HA fractions increases steadily, with decreasing wavelength, while that of the FA fraction begins to rise much more sharply and steeply. The differences between the self-extracted samples and the IHSS samples are not significant.

Various ratios of absorbance values measured at different wavelengths can be calculated. The main ratios such as E4/E6, E2/E3, and URI indexes are summarized in Table 3, from which some characteristics of the fractions can be deduced. The E4/E6 ratio is considered to be inversely proportional to the degree of condensation and aromaticity of the HSs and to the degree of humification [25,29].

The E2/E3 ratio correlates with the molecular size [33,34], similar to the E4/E6 ratio. The E2/E3 ratio for HAs is usually <5.0, whereas that of the FAs range from 6.0 to 8.5 [35,36]. The UV absorbance ratio index values (URI) provide information on the relative proportions between UV-absorbing functional groups and unsaturated bonds such as sp^2^-hybridized carbon atoms in aromatic rings.

The E4/E6 ratios of the HA samples in Table 3 are 3.1–5.5, which correlates with the literature data [29,33,34,35,36], indicating that HA fractions have larger molecules, better quality, stable structure, and proper humification degree.

The E4/E6 ratios of the FA samples are between 12.0 and 15.5, which also conforms with the literature data [29,33,34,35,36]. This suggests that the FA fractions are composed of smaller molecules than the HA and HY fractions. The E4/E6 ratios of the HY samples are 10.7 and 10.2, which allows us to say that the HY fraction molecular size is between HA and FA.

Similar conclusions can be drawn from the E2/E3 ratios. The E2/E3 ratios of the HA samples were 2.3–2.7, and since this characteristic parameter inversely correlates with the molecular size in the same way as the E4/E6 ratio, this confirms that the HA samples are composed of large molecules. The E2/E3 ratios of the FA samples were 4.4–5.4, confirming that the FA samples consist of smaller molecules than the HA samples. The E2/E3 ratios of the HY samples were 4.7–4.8, somewhat in between those of HA and FA, so the molecular size of HY is located between the HA and FA samples, which is consistent with the finding from the E4/E6 ratio.

Evaluating the URI ratio, the smallest values (1.2–1.4) belong to the HA fractions. HA fractions have the least amount of UV absorbent functional groups and/or unsaturated carbon bonds of all the fractions. So, the density of the functional groups and/or unsaturated bonds is the smallest in the HA fraction, since the URI ratio is directly proportional to the UV-absorbing functional groups and bonds (and their density). With values of 1.6 and 1.7, the HY fractions are again between HA and FA. Furthermore, again, FA has the highest ratio between 1.5 and 2.1 for the three factions of HS. These measured values agree with that found in the literature [25,29,33,34,35,36].

### 2.5. Free Radical Scavenging Activity Tested by the DPPH Method

The experimental data of the inhibitory effect of the different HS fractions on the DPPH radical decomposition are shown in Table 4. The data of the DPPH tests evidently show that the prepared materials have radical scavenging activity, because they effectively inhibited the decomposition of the DPPH radical.

As we mentioned before, the self-absorption of the samples at 517 nm (see UV–Vis spectra in Figure 2), where the absorbance of the purple DPPH radical is measured, causes confusion, and makes the measurements false; therefore, we had to make a correction as suggested by *Celiz* et al. [37]. We measured not only the samples according to the assay, but also the light absorption of HS solutions at 517 nm at the same concentrations present in the test mixtures. The self-absorbance values were subtracted from the absorbance measured in the DPPH assay to eliminate the disturbing effect of color sample solutions. The inhibition % values were calculated according to Equation (4) for the corrected absorbance data. In this way, we could obtain more realistic values. The IC_50_ values, i.e., HS concentrations corresponding to 50% inhibition for the different fractions are summarized in Table 4. 

If we compare the IHSS samples with our fractions extracted from leonardite, we can say that each fraction has an antioxidant effect, but the really clean IHSS HA fractions are the most effective. It can be assumed that the difficult and precise purification steps in the IHSS extraction protocol guaranteed the removal of many other substances that could reduce antioxidant efficiency in the inhibition of DPPH radical decomposition. The clean IHSS fractions are much more reactive and accessible to the DPPH radical. According to the DPPH test, they show better reducing ability than the fractions we extracted.

From the results of IHSS fractions, we can see that the strongest antioxidant effect belongs to the HA fractions, and there is little difference between the fractions extracted from peat and leonardite in favor of HA extracted from the latter. The IHSS FAP also shows an antioxidant effect, but is not nearly as powerful as the HA fractions.

Our samples also have radical scavenging activity. The HY fractions have the strongest antioxidant propensity, followed by the HA fractions, and then the FA fractions. In general, we can say that the alkaline extracted samples show greater antioxidant efficiency than the acid extracted fractions. 

Comparing our results with the literature data of *Phongpaichit* et al. [38], we can say that the IHSS HA fractions show intermediate free radical scavenging activity, because their IC_50_ value is between 50 and 100 µg/mL. The other samples, having IC_50_ values between 100 and 350, according to *Phongpaichit* et al. [38] are considered weak antioxidants. Recently, it was found by [39] that FA extract has a similar DPPH radical scavenging ability to ascorbic acid, and reached the inhibition of ~50% at a concentration of 1000 µg/mL. In the case of natural organic matter (FA and HA fractions) extracted from cretaceous shales, an antioxidant efficiency similar to that of ascorbic acid was also observed in the concentration range of 25–1000 µg/mL [40]. However, ~50% inhibition of DPPH radical decomposition was already achieved at a much lower concentration (~250 µg/mL) than above. The latter is almost the same as the average of the corrected LC50 values for our HS samples in Table 4. However, if we compare these IC_50_ values with other literature data of well-known antioxidants such as blueberry, raspberry, or blackberry [41], we can say our samples are as good or even better than these antioxidants. However, HS samples often do not reach the antioxidant efficiency of ascorbic acid, purple pitanga, and strawberry guava [42,43].

Based on the results above, we can prepare a sequence of our fractions extracted from leonardite, namely, the HY has the strongest radical scavenging activity followed by the HA and, finally, the FA fractions.

## 3. Materials and Methods

There are different methods for the extraction of HSs from different raw materials such as peat and leonardite [44,45]. As we mentioned above, we wanted to produce fractions that can be extracted on a larger, industrial scale. We also would like to see the differences between the alkaline and the acidic extracted HSs fractions in the respect of the DPPH method. The IHSS standard samples are a really clean fraction of the HSs, but producing them is a really difficult and costly process. To compare our self-extracted samples, we also bought IHSS samples, which are the closest to our fractions. Unfortunately, not all fractions are available from leonhardite at the IHSS, also the HY fraction is not available at the IHSS. So, we bought IHSS HA, which is derived from peat (IHSS HAP, 1S103H) and leonardite (IHSS HAL, 1S104H). In addition, we bought the IHSS FA (IHSS FAP, 2S103F), which is extracted from peat.

### 3.1. HS Samples Extracted from Leonardite

#### 3.1.1. Physical Preparation of Leonardite Sample

A bulk leonardite sample was collected in the Dudar coal mine, Hungary. For the experimental work, about 5 kg of each sample was obtained. First, we dry the leonardite samples, grind them, and sieve to obtain fractions between 0.5 and 3 mm. The prepared samples were sealed in plastic bags until use.

The stock solutions were freshly prepared. The laboratory grade concentrated HCl (Merck, Kenilworth, NJ, USA) was used to make a 20% (*w*/*w* %) working solution of HCl. The KOH solutions were prepared from 98% analytical grade KOH (Merck, Kenilworth, NJ, USA) in the concentration of 2.5% (*w*/*w* %). We also used ethanol at a concentration of 96% (*v*/*v*) (Merck, Kenilworth, NJ, USA). Laboratory grade concentrated H_2_SO_4_ (Merck, Kenilworth, NJ, USA) was used to make a 10% (*w*/*w* %) working solution of H_2_SO_4_. H_2_O_2_ solution was purchased in concentration 35 (*w*/*w* %) from Merck, Kenilworth, NJ, USA.

For extraction of HA, FA, and HY, the raw leonardite was chemically pretreated.

#### 3.1.2. Acidic Pretreatment by HCl

The leonardite was first digested with HCl to release Ca-bound organic materials. A 100 g was mixed with 500 mL of 20% HCl solution in a round flask. The content of the flask was carefully stirred for 2 h at 30–40 °C with a reflux condenser. The content was then filtered and washed to remove unreacted acid, dried in an oven at 40 °C, and preserved before the further steps.

#### 3.1.3. Fractionation of Acid Pretreated Leonardite

The acid pretreated sample was oxidized with hydrogen peroxide. We prepared a 10% suspension from 40 g of dried sample and then we mixed it with H_2_O_2_ solution (35 *w*/*w* %) in a 1:1 ratio (40 g). After adding all the H_2_O_2_ we boiled the suspension until the foaming stopped, to be sure there is no more excess H_2_O_2_ in the solution. The next step was centrifugations (4000 rpm for 5 min) and filtration with 8–12 µm filter paper. The ac-FA fraction was obtained from the filtrate.

After filtration, the sludge was subjected to alcoholic extraction with 96% ethanol to obtain ac-HA and ac-HY fractions. Then, 10 g of sludge was extracted with 90 g ethanol (96 *w*/*w* %) for an hour. The extraction was followed by sedimentation and filtration with 8–12 µm filter paper. The ac-HA was obtained from the sludge and the ac-HY from the filtrate. Finally, all fractions were dried in an oven at 40 °C and preserved for further experiments.

#### 3.1.4. Alkaline Pretreatment by KOH

The leonardite was treated with 2.5% KOH solution, then 500 g of leonardite was mixed with 1500 mL of 2.5% KOH solution in a glass reactor. The suspension was stirred for 2 h at 30–40 °C, a reflux condenser was used to avoid liquid loss. Then the content was centrifugated (4000 rpm for 5 min) to separate the sludge from the solution.

#### 3.1.5. Alkaline Fractionation

First, the supernatant was acidified to separate the al-FA and al-HA fractions. Then, 500 g of supernatant was taken and mixed with 100 g of 10 *w*/*w* % H_2_SO_4_. Due to a significant decrease in pH, the al-HA precipitated. The acidified suspension was filtered. The al-FA fraction was obtained from the filtrate and the al-HA fraction from the sludge after alcoholic extraction.

The filtered sludge was extracted with 96% ethanol to separate the al-HA from the al-HY. Then, 10 g of the sludge was taken and 90 g ethanol (96 *w*/*w* %) was added, and the suspension was extracted for an hour. The extraction was followed by sedimentation and filtration. The solid part was the al-HA and the filtrate was the al-HY. Finally, all the fractions were dried in an oven at 40 °C and stored for further experiments.

### 3.2. Elemental Analysis

Elemental compositions of the HSs were determined using a Fisons EA-1108 CHNS-O Element Analyzer (Thermo Fisher Scientific, Waltham, MA, USA) at University of Naples Federico II. The amounts of total carbon, nitrogen, and sulfur were determined. Roughly 2–5 mg of the sample was measured on a precision balance and mixed with an oxidizer (vanadium pentoxide [V_2_O_5_]) in a tin capsule. The container and the sample were combusted in a reactor at 1000 °C, and, after melting, the tin container promotes a violent reaction in the oxygen-enriched atmosphere. The products of this reaction (CO_2_, SO_2_, NO_2_) are carried by the carrier gas (helium) to the packed columns. The measured signal was quantified with a thermal conductivity detector (calibrated against pre-analyzed standards), and the elemental content of the sample was calculated in weight percentage. We corrected the measured data for moisture and ash content, and we calculated the oxygen content [30].

### 3.3. UV–Vis Spectroscopy

UV–Vis spectra were obtained on an SP-UV1100, DLAb spectrophotometer (DLAB Scientific Co., Ltd., Beijing, China) recording the absorption spectra between 190 and 800 nm. Dilute solutions of samples were measured in a 1 cm quartz cuvette. Humic constituents generally show strong absorbance in the UV–VIS range (190–800 nm), especially in the UV range, due to the presence of aromatic chromophores and/or organic constituents [46]. The UV–Vis spectra are commonly used to determine different empirical parameters characteristics of the quality, and the aromatic content of organic matter [46]. One of these is the E4/E6 ratio (also known as the “color ratio”). E4/E6 ratio is an internationally accepted parameter varied characteristically with humus quality. It is the ratio of extinction (E4/E6) measured at a wavelength of 465 and 665 nm. If the E4/E6 is high (7–8 or higher), the relatively small molecular, less stable FA and HA dominate, but if the ratio is between 3 and 5, then the larger molecular, better-quality HA predominate [47]. The E4/E6 value is related to the molecular size of HSs [48]. 

This ratio is independent of the sample concentration, but it well characterizes the difference between organic matter fractions and humus components of different origins. As the length of the condensation chain and the molecular size increase, the absorption signal shifts towards higher wavelengths (bathochromic shift) [49,50]. The E4/E6 ratio of HA and FA can be calculated:

(1) E4E6 ratio=Abs 465 nmAbs 665 nm

We also determined the E2/E3 ratio. Based on the ratio, we can infer the molecular size and degree of polymerization of dissolved organic matter (DOM). E2/E3 values are inversely proportional to polymerization and molecular weight.

(2) E2E3 ratio=Abs 250 nmAbs 365 nm

The third ratio is the UV absorbance ratio index (URI/UV absorption ratio) ratio, and it shows the ratio of UV-absorbing functional groups and unsaturated moieties. From this, we can infer the density of functional groups; the greater the density, the higher the value of the URI. More precisely, HA has the lowest URI value (1.59 for a HA, highest aromaticity), FA has a medium value (1.88 for a FA, intermediate aromaticity), and compounds with the lowest aromaticity (e.g., proteins) have the maximum value (13.50 for BSA, lowest aromaticity) [51].

(3) $$\mathrm{URI}\;\mathrm{ratio}=\frac{\mathrm{Abs}\;210\;\mathrm{nm}}{\mathrm{Abs}\;254\;\mathrm{nm}}$$

The UV–Vis absorption data were also used to correct the absorbance of the purple DPPH radical measured at 517 nm (see later) by its own light absorption of the HS fractions.

### 3.4. FT-IR Spectroscopy 

FT-IR spectra were recorded using the Diffuse Reflectance Infrared Fourier Transform (DRIFT) technique by means of a Perkin Elmer Frontier FT-IR/NIR spectrometer (Perkin Elmer, Waltham, MA, USA).

Approximately 2 mg of powder was weighed and ground with 200 mg of KBr in an agate mortar and then transferred to the sample holder cup. KBr and samples must be perfectly dry (since water can also be measured by IR, and then we would obtain false peaks, which might mask the peaks of other functional groups), so they must be dried at 105 °C. The homogeneous pale gray samples and pure KBr were placed in the sample holder, and then placed in the device. First, the spectrum of KBr was measured, and then the given sample was scanned. All spectra were recorded over the range of 4000–600 cm^−1^ at a resolution of 1 cm^−1^.

### 3.5. Free Radical Scavenging Activity Determination by DPPH Method

One of the earliest methods for measuring antioxidant activity is based on the radical scavenging ability of the stable DPPH molecule (2,2-Diphenyl-1-picrylhydrazyl, Merck, Kenilworth, NJ, USA). During the reaction, the dark purple free radical formed in the reaction mixture loses its color when it encounters an antioxidant molecule. This color change can be easily tracked. This is a popular method because DPPH, as a radical forming molecule, is stable, easily available, and less reactive. However, its big disadvantage is that it uses a stable radical, which cannot be produced in the normal metabolic processes of a living organism. Thus, this method cannot describe the effectiveness of the sample as an antioxidant of biological radicals [52]. During the measurement, 6 mL of DPPH working solution (0.1 mg/mL) was added to 1 mL of the sample, vortexed, and stored in completely dark for 30 min, to ensure the color reaction developed. After the 30 min passed, the absorbance was measured at 517 nm. After background correction, which was performed by measuring their own light absorption of each sample at 517 nm and subtracting this value from the absorbance value measured according to the method, we continued the calculation with the absorbance value corrected in this way [37]. The inhibition % (Inhib. %) and the IC_50_ value (the value corresponding to 50% inhibition) were calculated from the corrected absorbance data. As a function of the concentration, we plotted the calculated Inhib. % graphically, and using the curve fitting option of Microsoft Excel, the concentration value corresponding to 50% inhibition was calculated from the equation of the resulting curve.

(4) $$\mathrm{inhibition}\;\%=\frac{{\mathrm{Abs}}_{\mathrm{control}}-{\mathrm{Abs}}_{\mathrm{sample}}}{{\mathrm{Abs}}_{\mathrm{control}}}*100$$

Abs_control_ = absorbance of DPPH blank solution alone
Abs_sample_ = absorbance of DPPH test solutions together with the extracts of different concentrations, corrected by their own absorption.

As a reference, we used gallic acid (Perkin Elmer, Waltham, MA, USA). Gallic acid stock solution in 96% EtOH was prepared at a concentration of 1.0 mg/mL. A dilution series (0.2, 0.4, 0.6, 0.8, and 1.0 mg/mL concentration of gallic acid) was prepared from the stock solution and then measured as described above.

## 4. Conclusions

Our assumption that the extraction method affects not only the physico-chemical properties but also the free radical scavenging ability of the fractions has been proven. The experimental data have shown that the different fractionation methods affected, for example, the elemental composition, which is related to the content of the functional group of the fractions, their quality and quantity, and the FT-IR spectral characteristics, as well as the characteristics of their own UV–Vis light absorption and its effect on the free radical scavenging activity measured via the color change of purple DPPH radical. Furthermore, how do these physico-chemical differences affect the free radical scavenging activity of the fractions using the DPPH method? Our main observation from the antioxidant testing using the DPPH protocol is that the IHSS HA fractions have the strongest antioxidant effect regardless of whether their raw material is leonardite or peat. We should note that thorough cleaning of the IHSS samples likely contributes to this result. It is true that there is a difference between the antioxidant effect of samples extracted from leonardite and peat, but not nearly as large as the effect of the different extraction methods as we experienced in the case of our acidic- and alkaline-extracted samples. There are stereochemical constraints and differences in how the molecules of one fraction react with another antioxidant test molecule and how they react with each other, and different types of antioxidant-measuring methods should be applied to reveal the antioxidant potential of HS fractions. In this way, we can obtain a more comprehensive picture of the antioxidant effect of HSs.

Perhaps our most interesting finding was that all HA samples had higher antioxidant potential than FA samples. Although this is contrary to the results obtained by Klein et al. [19] for HS samples extracted from different sources, it has an economic advantage for the future use of HA as an antioxidant, since generally higher amounts of HA can be extracted from the raw materials than FA. Unfortunately, however, the industrial-scale production method of HS fractions cannot provide pure samples with higher antioxidant potential, such as IHSS HA samples.

## Figures and Tables

**Table 1:** The names, the short names, extraction method, and the raw material of the samples.

Name of the Fraction	Short Name (Code)	Extraction Method	Raw Material
IHSS Humic acid	IHSS HAP (1S103H)	IHSS	Peat
IHSS Humic acid	IHSS HAL (1S104H)	IHSS	Leonardite
IHSS Fulvic acid	IHSS FAP (2S103F)	IHSS	Peat
Humic acid	al-HA	Alkaline	Leonardite
Fulvic acid	al-FA	Alkaline	Leonardite
Himatomelanic acid	al-HY	Alkaline	Leonardite
Humic acid	ac-HA	Acidified	Leonardite
Fulvic acid	ac-FA	Acidified	Leonardite
Himatomelanic acid	ac-HY	Acidified	Leonardite

**Table 2:** The results of the elemental analysis of the samples. Where the C, H, N, O, and S are the mass content, and the different atomic ratios are shown to compare self-extracted samples to IHSS samples.

Sample	Elemental Composition (wt%)					Atomic Ratios		
	C	H	N	S	O_diff_	H/C	O/C	C/N
Leonardite	61.79 ± 0.15	6.95 ± 0.02	1.68 ± 0.04	1.22 ± 0.03	28.38	1.34	0.34	42.89
ac-HA	49.11 ± 1.41	5.61 ± 0.12	1.65 ± 0.04	0.85 ± 0.06	42.78	1.36	0.65	34.71
ac-FA	40.19 ± 0.12	5.38 ± 0.09	4.02 ± 0.01	2.97 ± 0.01	47.44	1.60	0.89	11.66
ac-HY	38.21 ± 1.23	5.20 ± 0.2	0.96 ± 0.06	1.11 ± 0.58	54.52	1.62	1.07	46.42
al-HA	45.03 ± 1.33	6.17 ± 0.19	0.70 ± 0.03	0.38 ± 0.03	47.72	1.63	0.80	75.02
al-FA	40.70 ± 1.17	5.37 ± 0.03	0.98 ± 0.05	1.30 ± 0.56	51.65	1.57	0.95	48.43
al-HY	38.43 ± 0.97	4.2 ± 0.5	1.78 ± 0.09	2.31 ± 1.01	53.28	1.30	1.04	25.18
IHSS HAP	56.37	3.82	3.69	0.71	37.34	0.81	0.50	17.82
IHSS HAL	63.81	3.7	1.23	0.76	31.27	0.69	0.37	60.50
IHSS FAP	51.31	3.53	2.34	0.76	43.32	0.82	0.63	25.57

**Table 3:** Spectrophotometric indicators calculated as the ratio of absorbance values at different wavelengths of HS fractions from leonardite for comparison with IHSS samples.

	E4/E6 Ratio ^1^	E2/E3 Ratio ^1^	URI Ratio ^1^
ac-HA	3.1	2.3	1.3
ac-FA	12.0	6.8	2.0
ac-HY	10.7	4.7	1.6
al-HA	4.2	2.5	1.2
al-FA	13.3	5.4	2.1
al-HY	10.2	4.8	1.7
IHSS HAP	5.5	2.7	1.4
IHSS HAL	5.3	2.5	1.3
IHSS FAP	15.5	4.4	1.5

^1^ E4/E6 = Abs_465 nm_/Abs_665 nm_; E2/E3 = Abs_250 nm_/Abs_365 nm_; URI = Abs_210 nm_/Abs_254 nm_.

**Table 4:** DPPH free radical scavenging activity of different HS fractions expressed as IC_50_ values.

	IC_50_ Values ^2^ µg/mL
ac-HA	260
ac-FA	400
ac-HY	200
al-HA	180
al-FA	310
al-HY	190
IHSS HAL	57
IHSS HAP	53
IHSS FAP	326

^2^ IC_50_ is the effective HS concentration at which DPPH radicals were scavenged by 50%.

## Data Availability

Not applicable.
